# Supplementary material for: ProteinShader: illustrative rendering of macromolecules
Source: BMC Struct Biol. 2009 Mar 30;9:19. doi: 10.1186/1472-6807-9-19 (PMC2672931; doi:10.1186/1472-6807-9-19)
Supplement: Additional file 1 — ProteinShader program without source code. This compressed file contains the complete ProteinShader program including associated libraries, but no source code. A README.txt file gives an overview of the ProteinShader distribution, and the index.html file in the help subdirectory has directions on getting started with the program as well as a set of tutorials. [file 1472-6807-9-19-S1.zip › ProteinShader-beta-0_9_4-binary/help/api/org/proteinshader/structure/class-use/Heterogen.html]

Uses of Class org.proteinshader.structure.Heterogen (ProteinShader API)


|  |  |  |
| --- | --- | --- |
| **Overview** \| **Package** \| **Class** \| **Use** \| **Tree** \| **Deprecated** \| **Index** \| **Help** | |  |
| PREV   NEXT | **FRAMES**    **NO FRAMES**     **All Classes** |  |


---


## **Uses of Class org.proteinshader.structure.Heterogen**

| Packages that use Heterogen | |
| --- | --- |
| **org.proteinshader.structure** | Holds the classes that store information from a Protein Data Bank file: Structure, Model, Chain, AminoAcid, Heterogen, Water, Atom, Bond, Helix, BetaStrand, Loop, *etc*. |
| **org.proteinshader.structure.visitor** | Holds Visitor classes that know how to traverse the hierarchy of objects held by class Structure. |

Uses of Heterogen in org.proteinshader.structure

| Methods in org.proteinshader.structure that return Heterogen | |
| --- | --- |
| `Heterogen` | `Chain.addNewHeterogen(String name, String residueID)`             Creates a new Heterogen with the name and Residue ID given as arguments, adds the Heterogen to the Chain's collection of Heterogens, and returns a reference to the new Heterogen. |
| `Heterogen` | `Chain.getHeterogen(String residueID)`             Returns the Heterogen with the residueID given as an argument. |

| Methods in org.proteinshader.structure that return types with arguments of type Heterogen | |
| --- | --- |
| `Iterator<Heterogen>` | `Chain.iteratorHeterogens()`             Returns an Iterator for the Heterogens held by this Chain. |

Uses of Heterogen in org.proteinshader.structure.visitor

| Methods in org.proteinshader.structure.visitor with parameters of type Heterogen | |
| --- | --- |
| `void` | `Visitor.visit(Heterogen heterogen)`             Traverses all Atoms owned by the Heterogen. |
| `void` | `SFWriterVisitor.visit(Heterogen heterogen)`             Writes all information held by a Heterogen. |
| `void` | `BondGeneratorVisitor.visit(Heterogen heterogen)`             NO OPERATION: This method is not needed because non-water heterogen bonds are given explicitly in the PDB structure entry. |

---


|  |  |  |
| --- | --- | --- |
| **Overview** \| **Package** \| **Class** \| **Use** \| **Tree** \| **Deprecated** \| **Index** \| **Help** | |  |
| PREV   NEXT | **FRAMES**    **NO FRAMES**     **All Classes** |  |


---

# *Copyright © 2007-2008*
